# Differences in Drug-Susceptibility Patterns between *Mycobacterium avium*, *Mycobacterium intracellulare*, and *Mycobacterium chimaera* Clinical Isolates: Prospective 8.5-Year Analysis by Three Laboratories

**DOI:** 10.3390/antibiotics12010064

**Published:** 2022-12-29

**Authors:** Mariana Fernandez-Pittol, Sara Batista-Arnau, Angely Román, Lorena San Nicolás, Laura Oliver, Olga González-Moreno, José Antonio Martínez, Rosanel Amaro-Rodríguez, Néstor Soler, Amadeu Gené, Araceli González-Cuevas, Griselda Tudó, Julian Gonzalez-Martin

**Affiliations:** 1 Servei de Microbiologia, CDB, Hospital Clínic de Barcelona, c/Villarroel 170, 08036 Barcelona, Spain; 2 ISGLOBAL, Institute for Global Health, c/Rosselló 132, 08036 Barcelona, Spain; 3 Departament de Fonaments Clínics, Facultat de Medicina i Ciències de la Salut, Universitat de Barcelona, c/Casanova 143, 080036 Barcelona, Spain; 4 SYNLAB Diagnósticos Globales, Departamento de Microbiología y Parasitología, 08950 Esplugues de Llobregat, Spain; 5 Servei de Malalties Infeccioses, Hospital Clínic-Universitat de Barcelona, 08036 Barcelona, Spain; 6 CIBER of Infectious Diseases (CIBERINFEC), Instituto de Salud Carlos III, 28029 Madrid, Spain; 7 Department of Pneumonology, Hospital Clínic-Universitat de Barcelona, 08036 Barcelona, Spain; 8 Laboratori, Hospital Sant Joan de Deu, 08950 Esplugues de Llobregat, Spain

**Keywords:** AST, MAC-complex, MIC-values, susceptibility profiles

## Abstract

**Background**: It has been suggested that *Mycobacterium avium*, *Mycobacterium intracellulare*, and *M. chimaera* have differential drug susceptibility patterns. We prospectively analyzed and compared the drug susceptibility patterns among these species over an 8.5-year period. **Methods**: A microdilution method (Slomyco^®^) was performed for drug susceptibility testing of 402 *M. avium*, 273 *M. intracellulare*, and 139 *M. chimaera* clinical isolates. **Results**: *M. avium* showed significantly higher resistance to moxifloxacin, ciprofloxacin, rifampicin, ethambutol, streptomycin, linezolid, cotrimoxazole, and clarithromycin. *M. avium* also showed higher minimum inhibitory concentrations (MIC) than *M. intracellulare* and *M. chimaera* against all drugs except ethionamide, to which *M. intracellulare* and *M. chimaera* showed greater resistance. **Conclusions**: Our series demonstrated differential drug resistance patterns among the most frequent *M. avium* complex species. *M. avium* was more resistant than *M. intracellulare* and *M. chimaera* versus eight antibiotics and showed greater MIC values to most of the antibiotics studied. These data suggest that knowledge of the local distribution and susceptibility profiles of these pathogens is essential for adequate clinical management.

## 1. Introduction

The incidence of non-tuberculous mycobacteria (NTM) infections is increasing. These infections mainly affect patients with inflammatory lung disease such as chronic obstructive pulmonary disease, bronchiectasis, or patients with immunodeficiency status [1,2]. *Mycobacterium avium* complex (MAC) and *M. abscessus* complex are the most frequent species isolated [2]. MAC include several species and subspecies, the most important being *Mycobacterium avium*, *Mycobacterium intracellulare*, and *Mycobacterium chimaera* [3]. These species are environmental microorganisms that can be isolated from clinical samples but are not always related to infection. The management of these patients is difficult since the infection is often associated with chronic pulmonary diseases and in many cases show scarce response to combined antibiotic therapy [4]. The recent treatment guidelines propose empirical antibiotic combinations based on in vitro susceptibility to macrolides, with a duration determined by clinical response and evolution [5,6] resulting in relapses and a trend to chronicity [4]. The differential geographic distribution and diversity of NTM has been described to understand the emerging impact of these opportunistic pathogens. MAC are the most frequent species isolated in the world, although the frequency varies among countries [7]. Among the species included in MAC, *M. avium* is the most frequently isolated in Europe, North and South America, while *M. intracellulare* is more predominant in South Africa, Asia, and Australia [7]. In recent years, the species *M. chimaera*, which is genetically very close to *M. intracellulare* [3], has been associated with the development of infection following open heart surgery. It has been demonstrated that these infections were linked to contaminated heater–cooler devices used during surgery in the United States and several European countries [8,9]. The infections were associated with the heater–cooler model 3T HCD (produced by Sorin, Germany) where the device was contaminated with *M. chimaera* at the manufacturing site until September 2014 [10]. Several reports suggest that *M. chimaera* is less virulent than the other two species [11,12]; however, data on the specific distribution and frequency of *M. chimaera* are scarce since most of the published epidemiological studies did not differentiate *M. chimaera* from *M. intracellulare*. Several publications have suggested that there is a differential drug susceptibility pattern between *M. avium* and *M. intracellulare* [13,14,15,16]. Therefore, these discrepancies are not clearly established and current guidelines [5,6] do not differentiate the treatment recommendations between these species. This further adds to the difficulty in managing these patients [11]. The present study aimed to compare the antimicrobial susceptibility patterns of *M. avium, M. intracellulare*, and *M. chimaera* in the clinical isolates from three clinical laboratories in the area of Barcelona during an 8.5-year period.

## 2. Results

A total of 814 MAC strains from 771 patients were analyzed; 402 (49.3%) were identified as *M. avium*, 273 (33.5%) as *M. intracellulare*, and 139 (17.0%) as *M. chimaera.* Of the samples, 527 (64.7%) were isolated from sputum, 223 (27.4%) from bronchoscopy samples, and 64 (7.8%) corresponded to samples of several origins such as lymph nodes, cerebrospinal fluid, pleural fluid, ascitic fluid, feces, blood, bone marrow, and synovial fluid. The distribution of samples did not differ among the three laboratories or among the three categories of species. A total of 477 (58.5%) samples were obtained from females and 337 (41.4%) from men. The median age of the cohort was 65 years (interquartile range 74.00–50.00). Table 1 shows the results of each drug according to the break point interpretation for each species. Statistically significant differences in resistance to seven drugs were observed between *M. avium* and *M. intracellulare* and between *M. avium* and *M. chimaera*, with *M. avium* showing greater resistance to ciprofloxacin, moxifloxacin, ethambutol, rifampicin, streptomycin, linezolid, and cotrimoxazole. Moreover, *M. avium* showed greater resistance to clarithromycin than *M. chimaera*. Comparative analysis between *M. intracellulare* and *M. chimaera* showed a similar susceptibility pattern between these species, except for two drugs, with which *M. intracellulare* showed statistically significant differences in resistance to clarithromycin (*p* = 0.05) and linezolid (*p* = 0.02). The distribution of the minimum inhibitory concentration (MIC) values differed for clarithromycin, ethambutol, cotrimoxazole, and ethionamide (Table 2). Overall, the *M. avium* strains showed higher MIC values than *M. intracellulare* and *M. chimaera* including the drugs used in regimes recommended for MAC pulmonary disease. There was also a trend to greater resistance of *M. intracellulare* and *M. chimaera* to ethionamide compared to *M. avium*.

## 3. Discussion

In the present study, we describe and compare the patterns of antimicrobial resistance of *M. avium, M. intracellulare*, and *M. chimaera* isolates to 12 antibiotics. The most important results were the differential resistance patterns for seven antibiotics, indicating that *M avium* is more resistant than *M. intracellulare* and *M. chimaera* to the quinolones ciprofloxacin and moxifloxacin and to rifampicin, ethambutol, linezolid, streptomycin, and cotrimoxazole. In addition, there were slight differences in resistance to clarithromycin and linezolid between *M. intracellulare* and *M. chimaera*.

The international guidelines for the treatment MAC-related pulmonary infections [5,6] recommend treatment with a macrolide-based regimen (clarithromycin or azithromycin) with rifampicin and ethambutol, without differentiating at a species level. Amikacin, quinolones, and other drugs can be used in cavitary or refractory clinical forms. However, several reports in the literature suggest different clinical outcomes between *M. avium* and *M. intracellulare*. A higher frequency of relapse has been associated with *M. avium* in the United States [11] and Japan [17], while in Korea [14,18], and in China [19], a more severe presentation and a worse prognosis has been described for *M. intracellulare*.

Regarding the drug susceptibility pattern, several studies published in recent years have compared the pattern of antibiotic resistance between *M. avium* and *M. intracellulare* [14,15,16,19,20,21,22]. A recent study by Maurer et al. [22] included isolates of four MAC species (*M. avium*, *M. intracellulare*, *M. chimaera*, and *M. colombiense*), concluding that there were no significant differences among these species. A study by Schulthess et al. [23] also suggested that *M. chimaera* has a drug pattern similar to other members of MAC. Although the methods, antibiotics studied, and the number of isolates included differed, *M. avium* showed a trend to being more resistant. The study of Cho et al. [14], developed in Korea, was an exception, finding *M. intracellulare* to be more resistant. This is remarkable, since it is the largest study published to date and includes two thousand isolates. With respect to the antibiotics used, there is a wide variation among studies. Amikacin, ethambutol, linezolid, and moxifloxacin are the antibiotics most associated with *M. avium* resistance [14,16,19,20,21]. In addition to antibiotics related to resistance, *M. avium* isolates showed a trend to presenting a higher MIC to most of the drugs studied, as shown in the present study. The only exception was the association between *M. intracellulare* and *M. chimaera* and resistance to ethionamide, which was observed in our study and described in other studies for *M. intracellulare* [16,24]. We also found a slight difference between *M. intracellulare* and *M. chimaera* with respect to clarithromycin. This difference was statistically significant, probably because we did not find any *M. chimaera* isolates resistant to clarithromycin. This is an issue that should be studied in the near future.

As shown in the previously mentioned studies, there were differences in the drug susceptibility patterns among these MAC species, especially between *M. avium* and the other two. The question is why a translation to clinical outcome is not clearly observed. A possible explanation is the importance of the resistance to the antibiotics used in the recommended treatment schedules. There is no doubt as to the main role of macrolides in the treatment of these infections, being the backbone of therapeutic schemes. In the studies analyzed, the percentage of resistance to clarithromycin was less than 5%, as in our study as well as in those suggesting differences in resistance to this drug among the MAC species. A similar situation is applicable to amikacin.

Differences in geographical distribution between the species have been described in Europe [7], with *M. avium* being predominant, although with important differences among countries. In Asian countries such as China and Korea, the *M. intracellulare* isolates predominate [14,15,16,19]. Moreover, in China, an increasing percentage of *M. intracellulare* isolates was observed between 2000 and 2019, being the most frequent species isolated together with *M. abscessus* [25]. Other reports in Asia have also described differences between these species depending on the drugs used, demonstrating a high variability in the behavior of MAC species [25,26,27]. In the present study, we found a slightly higher frequency of *M. avium* over *M. intracellulare* with 49.3% of isolates.

Although the specific reasons for geographic differences in species distribution are not well-known, some data should be taken into consideration. First, the worldwide distribution of MAC species, associated with potable water systems and natural water, with different environmental conditions between countries and geographical areas. Studies specifically aimed at determining the presence of MAC species in water have demonstrated a different environmental niche for *M. intracellulare*, being much more frequently isolated in biofilm than *M. avium* [28]. Second, genetic diversity has been described for *M. avium* isolates. A recent multicenter study including isolates from European and Asian countries found genetic diversity in *M. avium* isolates, suggesting that they may originate from different sources, routes of transmission, and perhaps clinical manifestations [29]. Along the same line, it has been suggested that mycobacterial grouping of *M. avium* based on variable number of tandem repeats typing techniques is associated with therapeutic response in lung infections [30]. Apart from genetic diversity, the rigorous identification of MAC species and subspecies could be interesting to elucidate the clinical significance of the MAC [31]. In general, all of these data show that the changes observed in MAC depend on the species, and probably subspecies and genotypes as well as the geographical area.

All of these results suggest the importance of species identification of the members of MAC and the need to study drug patterns in different geographic areas. In addition, studies should be performed to elucidate possible specific therapeutic approaches for the treatment of diseases caused by the different species of MAC.

## 4. Materials and Methods

### 4.1. Study Design

Differences in drug susceptibility patterns among the *M. avium*, *M. intracellulare*, and *M*. *chimaera* clinical isolates were analyzed over an 8.5-year period from January 2013 to June 2021. The analysis focused on 12 antibiotics.

### 4.2. Clinical Isolates

During the study period, all of the isolates identified as *M. avium, M. intracellulare*, and *M. chimaera* were included in the analysis. Most of the isolates included were obtained from cultures of clinical samples from patients with chronic pulmonary disease collected during diagnostic procedures or follow-up controls. Extra-respiratory samples were obtained from immunosuppressed patients with hematological malignancy or HIV infection or AIDS. Three laboratories participated in the collection and culture of the samples: The Microbiology Department of the Hospital Clinic of Barcelona (MDHC), the Microbiology Laboratory of SYNLAB Laboratories, and the Microbiology Laboratory of Hospital Sant Joan de Deu. Final identification and the drug susceptibility testing (DST) were centralized in the MDHC.

### 4.3. Microbiological Methods

Species identification and DST were performed prospectively. Mycobacterial culture was performed on solid Löwenstein–Jensen medium (Becton Dickinson, Franklin Lakes, NJ, USA) and in liquid BD BACTEC Mycobacteria Growth Indicator Tubes (MGIT, BACTEC 960 system, Becton Dickinson) according to the manufacturer’s instructions.

#### 4.3.1. Species Identification

The identification of the isolates was first performed using MALDI-TOF (Bruker, Bremen, Germany) following a previously described protocol [32]. Since *M. intracellulare* and *M. chimaera* cannot be differentiated using this MALDI-TOF procedure, the isolates identified as *M. intracellulare-chimaera* were submitted for sequencing of the 16S rRNA [33] and *rpo*B genes [34]. The *hsp*65 gene [35] was sequenced in the case of discrepancy. The fragments amplified were 564 bp (16S rRNA), 723 bp (*rpo*B), and 441 bp (*hsp*65). The primers used were as follows: for 16S rRNA, g2R (5′-GAGAATTCGTGCTTAACACATGCAAGTCG-3′) and M582R (5′-ATGGATCCGTGAGATTTCACGAACAACGC-3′); for *rpo*B, MycoF (5′-GGCAAGGTCACCCCGAAGGG-3′) and MycoR (5′-AGCGGCTGCTGGGTGATCATC-3′); for *hsp*65, Tb11 (5′-ACCAACGATGGTGTGTCCAT-3′) and Tb12 (5′-CTTGTCGAACCGCATACCCT-3′). PCR reactions were performed in DT lite 5 thermocycler (Certest Biotec SL, Zaragoza, Spain). The PCR mix contained 1.6 µL (5 mmol) of each pair of primers (Integrated DNA Technologies, San Diego, CA, USA), 10 µL of SensiFAST™ SYBR^®^ (Watlham, MA, USA), and 11.8 µL of the purified DNA. The PCR conditions were 2 min at 95 °C followed by 35 cycles of 95 °C for 15 s, 60 °C for 15 s, 72 °C for 30 s, and 78 cycles at 95 °C for 15 s. For PCR purification, first, a mixture with 4 µL of ExoSAP-IT™ (Applied Biosystem, Watlham, MA, USA) and 10 µL of the PCR product was processed in the thermocycler (Applied Biosystem) under the following conditions: 37 °C for 15 min and 85 °C for 15 min. Second, to complete the purification step, we mixed 4 µL of BigDie (Applied Biosystem), 2 µL of each pair of primers, and 4 µL of the DNA. The thermocycler conditions were 96 °C for 30 s, 50 °C for 15 s, and 60 °C for 4 min followed by 25 cycles. Finally, Sanger sequencing was used. For the sequencing reaction, we mixed 3.5 µL sterile water, 0.8 µL BigDie Terminator v3.1 Cycle Sequencing Kit (Applied Biosystem), 1.7 µL BigDie buffer, 0.5 µL M13 forward and reverse primers (Sigma-Aldrich, Sant Louis, MO, USA), and 3.5 µL of the amplification product. Purified amplicons were sequenced using the dye terminator cycle with the following profile: 96 °C for 1 min, followed by 30 cycles of 96 °C for 10 s, 50 °C for 5 s, and 60 °C 10 s. Purification of the sequencing reactions was performed with EdgeBio^®^ (San Jose, CA, USA) followed by the deionized formamide addition step to separate the sequencing reactions, and finally, capillary electrophoresis. Samples of water, as negative controls, were included in every round of sequencing. The sequences were compared to the following included in the GenBank database using the BLAST tool. For *M. chimaera*: 16S rRNA accession AJ548480.2, *rpo*B accession GQ153309.1, and *h*sp65 accession JF795578.1; for *M. intracellulare*: 16S rRNA accession X52927.1, *rpo*B accession GQ153307.1, and *hsp*65 accession AY299169.1.

#### 4.3.2. DST

DST was performed once for each patient suspected of having infection or being colonized using the first isolate identified as *M. avium* or *M. intracellulare-chimaera*. A second DST was conducted in the isolates from samples separated by at least six months from the first. The DST was performed using a commercial microdilution method Sensititre™ Myco SLOMYCOI AST plate (Thermo Fisher Scientific, Waltham, MA, USA) following the manufacturer’s instructions. Twelve antibiotics from the commercial panel were included in the analysis: clarithromycin, ciprofloxacin, streptomycin, doxycycline, ethionamide, rifabutin, ethambutol, moxifloxacin, rifampicin, amikacin, linezolid, and cotrimoxazole. The break points of resistance were established according to the CLSI guidelines [36]. For antibiotics not included in these guidelines, the break points used were based on the literature [37] (see Table 3).

#### 4.3.3. DST Quality Control

The reference strain *M. avium* ATCC 25291 was used as the quality control of DST. The strain was tested once a month throughout the study period.

### 4.4. Statistical Analysis

The frequency data were described by sex, sample type, and isolate identification considering the three species: *M. avium*, *M. intracellulare*, and *M. chimaera*. Categorical data are expressed as numbers and percentages. The Chi-square test was used to compare the susceptibility profile for each drug among the species according to the following comparisons: *M. avium* versus *M. intracellulare; M. avium* versus *M. chimaera*; *M. intracellulare* versus *M. chimaera*. For the statistical analysis (Table 1), intermediate values were excluded. All of the calculations were made using Rstudio package version 4.0.5.

## 5. Conclusions

In conclusion, our series demonstrated differential drug resistance patterns among the most frequent *M. avium* complex species. *M. avium* was more resistant than *M. intracellulare* and *M. chimaera* versus eight antibiotics and showed greater MIC values to most of the antibiotics studied. These data suggest that knowledge of the local distribution and susceptibility profiles of these pathogens is essential for adequate clinical management.

## Figures and Tables

**Table 1:** Distribution of the susceptibility testing results of 402 *M. avium*, 273 *M. intracellulare*, and 139 *M. chimaera* isolates versus 12 antibiotics.

	*M. avium* (*n* = 402)			*M. intracellulare* (*n* = 273)				*M. chimaera* (*n* = 139)			
ATB	S (%)	I (%)	R (%)	S (%)	I (%)	R (%)	*p* Value *	S (%)	I (%)	R (%)	*p* Value *
CLA	355 (87.0)	37 (8.7)	17 (4.2)	430 (95.6)	6 (1.0)	12 (3.2)	0.3	136 (97.8)	3 (2.1)	0 (0.0)	0.01
CIPRO	25 (6.2)	61 (15.1)	316 (78.6)	42 (15.3)	39 (14.2)	192 (70.3)	0.00001	16 (11.5)	25 (17.9)	98 (70.5)	0.03
STREP	-	-	389 (70.3)	-	-	164 (60.0)	0.00001	-	-	81 (58.2)	0.00001
DOXY	0 (0.0)	1 (0.2)	401 (99.7)	2 (0.7)	5 (1.8)	266 (97.4)	0.08	1 (0.7)	2 (1.4)	136 (97.8)	0.08
ETHI	-	-	92 (22.8)	-	-	113 (41.3)	2.8	-	-	58 (41.7)	1.8
RIB	381 (94.7)	-	21 (5.2)	266 (97.4)	-	7 (2.5)	0.08	136 (97.8)	-	3 (2.1)	0.1
EB	-	-	394 (98.0)	-	-	220 (80.5)	0.00001	-	-	115 (82.7)	0.00001
MOX	180 (44.7)	151 (37.5)	71 (17.6)	149 (54.5)	93 (34.0)	31 (11.3)	0.007	71 (51.0)	58 (41.7)	10 (7.1)	0.003
RIF	65 (16.1)	-	337 (83.8)	136 (49.8)	-	137 (50.1)	0.00001	72 (51.7)	-	67 (48.2)	0.00001
AK	387 (96.2)	8 (1.9)	7 (1.7)	271 (99.2)	0 (0.0)	2 (0.7)	0.2	137 (98.5)	2 (1.4)	0 (0.0)	0.1
LNZ	47 (11.6)	97 (24.1)	258 (64.1)	84 (30.7)	92 (33.6)	97 (35.5)	0.00001	47 (33.8)	63 (45.3)	29 (20.8)	0.00001
SXT	35 (8.7)	-	367 (91.2)	58 (21.2)	-	215 (78.7)	0.00001	21 (15.1)	-	118 (84.8)	0.03

ATB: antibiotic; CLA: clarithromycin; CIPRO: ciprofloxacin; STREP: streptomycin; DOXY: doxycycline; ETHI: ethionamide; RIB: rifabutin; EB: ethambutol; MOX: moxifloxacin; RIF: rifampicin; AK: amikacin; LNZ: linezolid; SXT: cotrimoxazole; S: susceptible; I: intermedium; R: resistance; (-): since there is no break point set for this/these categories by the Clinical and Laboratory Standards Institute (CLSI) guidelines, the number of isolates could not be specified * *p* values show the X^2^ test results between *M. avium* and *M. intracellulare* and between *M. avium* and *M. chimaera*.

**Table 2:** Range of the distribution of the minimum inhibitory concentration results of *M. avium*, *M. intracellulare*, and *M. chimaera* versus 12 antibiotics.

		*M. avium*	*M. intracellulare*	*M. chimaera*
	MIC	Total Isolates (%)	Total Isolates (%)	Total Isolates (%)
Clarithromycin	≤2	59 (14.6)	204 (74.7)	112 (80.5)
	4–8	296 (72.3)	57 (20.8)	24 (17.2)
	16	37 (8.7)	3 (1.0)	3 (2.1)
	≥32	17 (4.2)	9 (3.2)	0 (0.0)
Ethionamide	≤2.5	243 (60.4)	118 (43.2)	60 (43.1)
	4–10	89 (22.1)	53 (19.4)	34 (24.4)
	>10	70 (17.4)	102 (37.3)	45 (32.3)
Rifabutin	≤2	381 (94.7)	267 (97.8)	136 (97.8)
	4–8	18 (4.4)	4 (1.4)	3 (2.1)
	>8	3 (0.7)	2 (0.7)	0 (0.0)
Ethambutol	≤2.5	8 (1.9)	53 (19.4)	24 (17.2)
	4–8	241 (59.9)	164 (60.0)	62 (44.6)
	≥16	153 (38.0)	56 (20.5)	53 (38.1)
Moxifloxacin	≤1	332 (82.3)	242 (88.6)	129 (92.8)
	4–8	58 (14.3)	30 (10.9)	9 (6.4)
	>8	13 (3.2)	1 (0.3)	1 (0.7)
Rifampicin	≤1	66 (16.4)	136 (49.8)	72 (51.7)
	2–8	183 (45.5)	122 (44.6)	59 (42.4)
	>8	153 (38.0)	15 (5.4)	8 (5.7)
Amikacin	≤1	2 (0.4)	21 (7.6)	7 (5.0)
	2–16	385 (95.7)	250 (91.5)	130 (93.5)
	32	8 (1.9)	0 (0.0)	2 (1.4)
	≥64	7 (1.7)	2 (0.7)	0 (0.0)
Linezolid	≤8	47 (11.6)	84 (30.7)	47 (33.8)
	16	97 (24.1)	92 (33.6)	63 (45.3)
	≥32	258 (64.1)	97 (35.5)	29 (20.8)
Cotrimoxazole	≤2	35 (8.7)	58 (21.2)	21 (15.1)
	4–8	113 (28.1)	83 (30.4)	47 (33.8)
	>8	254 (63.1)	132 (48.3)	71 (51.0)
Ciprofloxacin	≤2	86 (21.3)	81 (29.6)	41 (29.4)
	4–8	152 (37.8)	136 (49.8)	71 (51.0)
	≥16	164 (40.7)	56 (20.5)	27 (19.4)
Streptomycin	≤2	3 (0.7)	40 (14.6)	16 (11.5)
	4–16	78 (19.4)	179 (65.5)	77 (55.3)
	>16	321 (79.8)	54 (19.7)	46 (33.0)
Doxycycline	≤1	0 (0.0)	2 (0.7)	1 (0.7)
	2–4	1 (0.2)	5 (1.8)	2 (1.4)
	≥8	401 (99.7)	266 (97.4)	136 (97.8)

MIC: minimum inhibitory concentration.

**Table 3:** Break point recommendations for the interpretation of antimicrobial susceptibility testing for slow-growing mycobacteria.

Antibiotic	Reference	MIC (µg/mL)		
		Susceptible	Intermediate	Resistant
Clarithromycin	CLSI [35]	≤8	16	≥32
Ciprofloxacin	CLSI [36]	≤1	2	≥4
Streptomycin	CLSI [35]	-	-	≥10
Doxycycline	CLSI [36]	≤1	2–4	≥8
Ethionamide	CLSI [35]	-	-	≥10 *
Rifabutin	CLSI [36]	≤2	-	≥4
Ethambutol	CLSI [35]	-	-	≥4
Moxifloxacin	CLSI [35]	≤1	2	≥4
Rifampicin	CLSI [36]	≤1	-	≥2
Amikacin	CLSI [35,36]	≤16	32	≥64
Linezolid	CLSI [35]	≤8	16	≥32
Cotrimoxazole	CLSI [36]	≤2/38	-	≥4/76

MIC: minimum inhibitory concentration; CLSI: Clinical and Laboratory Standards Institute; (-) No break point set by CLSI. * Data supporting equivalency with 7H11 (10.0 µg/mL) are limited.

## Data Availability

Not applicable.
